# Hydrogen Sensor Based on Tunable Diode Laser Absorption Spectroscopy

**DOI:** 10.3390/s19235313

**Published:** 2019-12-03

**Authors:** Viacheslav Avetisov, Ove Bjoroey, Junyang Wang, Peter Geiser, Ketil Gorm Paulsen

**Affiliations:** 1 NEO Monitors AS, Prost Stabels vei 22, N-2019 Skedsmokorset, Norway; ove.bjoroy@neomonitors.com (O.B.); ketil.paulsen@neomonitors.com (K.G.P.); 2 Yinian Sensors Technology Co., Ltd., Shenzhen 518126, China; junyang.wang@ecamana-tech.com

**Keywords:** gas sensor, hydrogen sensor, diode laser, TDLAS, WMS, absorption spectroscopy, laser spectroscopy, hydrogen

## Abstract

A laser-based hydrogen (H_2_) sensor using wavelength modulation spectroscopy (WMS) was developed for the contactless measurement of molecular hydrogen. The sensor uses a distributed feedback (DFB) laser to target the H_2_ quadrupole absorption line at 2121.8 nm. The H_2_ absorption line exhibited weak collisional broadening and strong collisional narrowing effects. Both effects were investigated by comparing measurements of the absorption linewidth with detailed models using different line profiles including collisional narrowing effects. The collisional broadening and narrowing parameters were determined for pure hydrogen as well as for hydrogen in nitrogen and air. The performance of the sensor was evaluated and the sensor applicability for H_2_ measurement in a range of 0–10 %v of H_2_ was demonstrated. A precision of 0.02 %v was achieved with 1 m of absorption pathlength (0.02 %v∙m) and 1 s of integration time. For the optimum averaging time of 20 s, precision of 0.005 %v∙m was achieved. A good linear relationship between H_2_ concentration and sensor response was observed. A simple and robust transmitter–receiver configuration of the sensor allows in situ installation in harsh industrial environments.

## 1. Introduction

An increased demand for hydrogen gas sensors is strongly coupled with the expanded use of hydrogen gas (H_2_) in industry [1,2]. Hydrogen is an important feedstock in many industrial processes and applications, including the oil and gas industry, chemical plants, and the steel industry, among others. Refineries use hydrogen in many operations (e.g., hydrotreating of various refinery process streams and hydrocracking of heavy hydrocarbons). Analyzing hydrogen in complex and varying gas mixtures is challenging, and measurements are normally performed using gas chromatographs that entail slow response times and high operational costs. Since hydrogen is highly flammable, strict regulations for using H_2_ safety sensors apply. Many different types of hydrogen safety sensors are commercially available [3], and the common principle is a sensing element that is altered (e.g., by resistance) when in contact with hydrogen. This mode of operation precludes the use of these point-type hydrogen sensors in reactive, corrosive, and/or dusty gas streams. For this reason, contactless hydrogen sensing is highly desired. Diode lasers are extremely attractive for this purpose due to their inherently low-intensity noise and narrow linewidth. These properties enable the highly selective and sensitive probing of narrow and extremely weak H_2_ absorption lines. As a diatomic homonuclear molecule, H_2_ has no dipole moment that could create strong optical absorption in the infrared region. The absorption spectrum of H_2_ is therefore limited to vibrational bands of very weak electric quadrupole transitions [4], which is why laser-based detection of H_2_ so far has been limited to extractive cavity-enhanced sensors based on cavity ring-down spectroscopy (CRDS) [5,6], intra-cavity output spectroscopy (ICOS) [7,8], and optical-feedback cavity-enhanced absorption spectroscopy (OF-CEAS) [9]. Common in these techniques is confinement of the laser light in a high-finesse optical cavity by using a set of highly reflective mirrors. Up to several kilometers of effective absorption pathlength can be obtained, which allows for detection of very weak hydrogen quadrupole transitions. However, for use in industrial applications, where extremely high sample purity can be difficult to achieve, contamination of mirrors is an issue. If not properly handled, this will lead to degradation of the sensor sensitivity and can ultimately damage the coatings of the high-reflectivity mirrors. For this reason, cavity-based H_2_ sensors often impose rigid requirements on gas sampling and conditioning systems and typically require periodic maintenance.

Tunable diode laser absorption spectroscopy (TDLAS) [10,11] is a sensitive and selective method that directly probes the process (in situ) without having to extract gas samples. Gas sensors based on TDLAS are frequently used for many industrial process-control, emission-monitoring, and safety applications, and are well accepted throughout many industries [12,13,14,15,16]. The underlying measurement principle is inherently contactless, and the instrumentation is consequently not exposed to potentially corrosive process gases. Concentration readings are made available in real time, which is ideal for fast and efficient process control and safety-related measurements. Furthermore, in situ measurements have low maintenance requirements and thus reduce the operational costs. In general, compared to cavity-enhanced techniques, TDLAS has less complexity and is more robust, which has made TDLAS-based sensors the preferred platform for many industrial process-control and safety applications.

In this paper, we present the first laser-based infrared hydrogen absorption sensor for in situ hydrogen measurements. The sensor can be reconfigured for extractive measurements for applications where in situ installation is not feasible due to, for example, high pressure and/or high temperature. Optical windows isolate the process gas from the sensor so that the advantage of contactless measurement is maintained. The presented hydrogen sensor is based on the commercial LaserGas II platform [17] manufactured by NEO Monitors AS. The instrument was used to study a selected hydrogen absorption line in terms of line broadening and narrowing effects. To validate the measurements, lineshape modelling using different spectral line profiles was performed. The sensor was capable of measuring hydrogen with a precision of 0.02 %v∙m for 1 s of integration time, which is better than most intended safety applications require (assuming a measurement range of 0–10 %v). Using a response time of 1 s, an estimated limit of detection (LOD) for H_2_ of 0.1 %v for 1 m of absorption pathlength was achieved.

## 2. Sensor Design

The developed sensor follows the classical in situ TDLAS design and consists of transmitter and receiver units. The transmitter unit contains a diode laser, collimating optics, a microprocessor board, and all input–output electronics. The transmitter unit also has a built-in cell for H_2_ validation. The receiver unit incorporates a photodetector, focusing optics, and signal detection electronics (amplifier, mixer, etc.). 

The sensor is based on the wavelength modulation spectroscopy (WMS) technique, which is well described in the literature [18,19,20]. This technique has been proven to be very useful in trace gas sensing due to its ability to perform very sensitive interference-free measurements directly in the process or across stacks without sample extraction and preconditioning. Since WMS provides nominally baseline-free absorption signals, it is especially suited for measuring weak absorbance. Recently published comparisons of WMS and direct absorption spectroscopy (DAS) techniques revealed that WMS is approximately one order of magnitude more sensitive [21,22,23]. 

**Figure 1:**
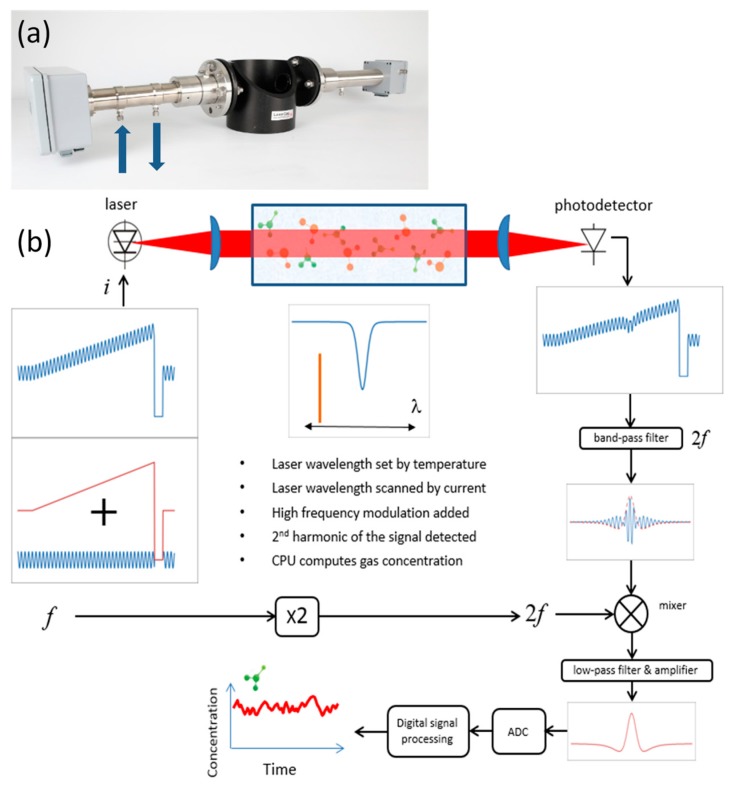
(**a**) Tunable diode laser absorption spectroscopy (TDLAS) H2 sensor mounted on a demo pipe. Transmitter unit is on the left and receiver unit on the right. Gas inlet and outlet of the built-in validation gas cell are indicated. (**b**) Schematic overview of the principles of sensor operation. A sinusoidally modulated current ramp is applied to the laser, which is swept in frequency across the transition of interest. After interacting with the sample, the absorption information is encoded in the transmitted intensity, which is measured using a photodetector. The photodetector signal is amplified, filtered, mixed, and digitized. Finally, digital signal processing is used to retrieve the concentration (and possibly other relevant parameters).

Figure 1a shows a photograph of the LaserGas II sensor mounted on the demo pipe using DN50 flanges, and Figure 1b depicts a schematic diagram and the basic principle of the sensor operation. 

A distributed feedback (DFB) diode laser from Nanoplus (Nanosystems and Technologies GmbH) emitting near 2122 nm was used in the sensor. The laser had an output power of about 5 mW at the driving conditions specified herein. The temperature of the diode laser was stabilized at around 30 °C with high accuracy (typically in the mK range) to set the average emission wavelength of the laser. A DC current of 70 mA was applied to operate the laser above its threshold, and a current ramp of 10 mA with a duration of about 2 ms was used to tune the laser about 0.4 cm^−1^ across the H2 absorption line of interest. The repetition rate of the current ramps was 150 Hz. After each ramp, the laser current was switched off for signal normalization purposes. A sinusoidal current of 2 mA amplitude and frequency f of 100 kHz was added to the current ramp in order to modulate the laser wavelength for WMS implementation. The collimated laser beam was directed through the target gas, captured by the receiver optics, and focused onto an InGaAs pin photodetector (Hamamatsu G12183-020K). The signal detected by the photodetector was bandpass-filtered to select the 2f component, and the filtered signal was detected using an analog mixer, a low-pass filter, and an amplifier. The bandwidth of the detected signal was 10 kHz. Further, the 2f WMS signal was digitized using an AD converter and normalized to the measured direct signal. Before calculating the gas concentration, additional digital signal processing was applied to improve the signal-to-noise ratio (SNR), which optionally could be digital filtering, wavelet denoising, or baseline fitting.

## 3. Line Selection

**Figure 2:**
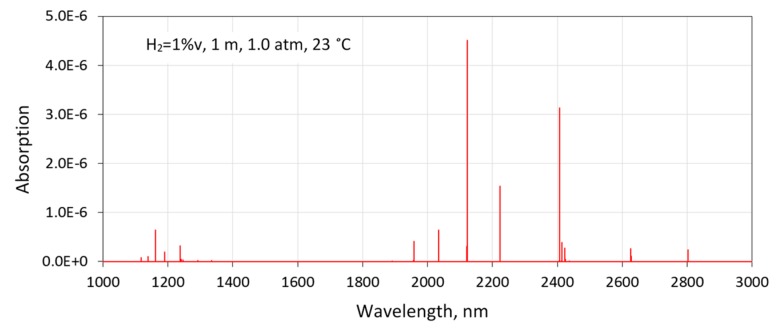
High-resolution transmission molecular absorption database (HITRAN) simulation of H_2_ absorption (1 %v∙m) using default air broadening parameters listed in the HITRAN2016 database.

The absorption spectrum of H_2_ in the infrared region was very sparse and extremely weak. Figure 2 shows a simulation of H_2_ absorption using default air broadening parameters listed in the high-resolution transmission molecular absorption database (HITRAN) [4]. The fundamental (1–0) vibrational electric–quadrupole transitions of H_2_ are between 1900 and 3000 nm. There are only a few lines visible due to the large rotational constant that follows from the low mass of the H_2_ molecule. The first overtone band (2–0) of H_2_ is located between 1100 and 1500 nm. Compared to the fundamental band, the overtones have been studied much more extensively using cavity-enhanced spectrometers [6,24].

**Figure 3:**
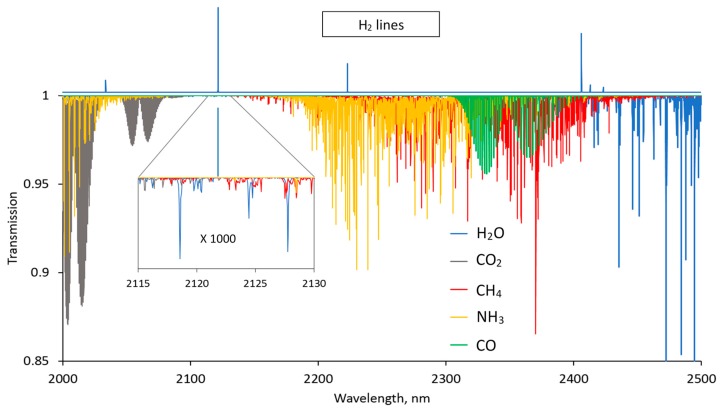
HITRAN simulation of transmission spectra of H_2_O, CO_2_ (1 %v∙m), CH_4_, NH_3_, and CO (0.1 %v∙m) and positions of the H_2_ lines. Inset: enlarged view of absorption spectra around the 2122-nm H_2_ line.

From the HITRAN simulations, three transitions in the fundamental vibrational band were identified as potentially suitable for H_2_ gas sensing: 2407 nm (4155.3 cm^−1^), 2223 nm (4497.8 cm^−1^), and 2122 nm (4712.9 cm^−1^). The best available transition for industrial applications is not necessarily the strongest, but the one with least interference from gases such as water vapor (H_2_O), carbon dioxide (CO_2_), methane (CH_4_), ammonia (NH_3_), and carbon monoxide (CO). Figure 3 shows the simulated transmission spectra of these gases for the wavelength region between 2000 nm and 2500 nm. The hydrogen spectrum (line positions) is depicted on top of the plotted transmission spectra. For demonstration purposes, H_2_O and CO_2_ concentrations were set to 1 %v, while CH_4_, NH_3_, and CO were set to 0.1 %v. The hydrogen transition at 2122 nm can be identified as potentially best suited for the sensor, since it is not only the strongest H_2_ line but also is located at the transmission window for the plotted spectra. However, all these gases except CO absorb in the close vicinity of this H_2_ line, which is demonstrated by the inset of Figure 3, showing a 1000 times magnified portion of the spectra around the 2122 nm line. Each of the identified H_2_ lines was investigated in more detail by a closer look into high-resolution simulated spectra. The H_2_ line at 2407 nm is close to the strong H_2_O absorption band at 2700 nm and several strong H_2_O lines overlap with the weak H_2_ line. Likewise, the line is within the spectral region of the strong CH_4_ absorption band. Also, CO and NH_3_ should be considered to be potentially interfering gases. It should be mentioned that the 2407 nm line is free of CO_2_ interference; nevertheless, using this line for H_2_ sensing is problematic due to interference with the other mentioned gases. The H_2_ line at 2223 nm is free of interference with CO_2_ and H_2_O (at least around ambient temperature), which makes it attractive for some applications. However, the line suffers from strong NH_3_ interference and, to a somewhat lesser extent, from CH_4_ interference as well. In addition, the strength of this line is about one-third the strength of the H_2_ line at 2122 nm. Since the line strength is a crucial factor for sensing hydrogen, the line at 2122 nm (S(1) (1–0) transition, 2121.8 nm, 4712.9 cm^−1^) was chosen. As mentioned, this line is not free of CH_4_ and NH_3_ interference, although the interference is much weaker than for the other two H_2_ lines. In addition, a weak CO_2_ absorption line relatively close to the H_2_ line (0.13 cm^−1^ apart) must be considered for applications with percentage levels of CO_2_. 

## 4. Experimental Results and Modelling of the H_2_ Lineshape

The line strength of the S(1) (1–0) H_2_ transition is 3.2 × 10^−26^ cm/molecule [4]. Simulations using an air broadening coefficient of 0.05 cm^−1^/atm (default value listed in HITRAN2016) and the Voigt profile indicated about 5 × 10^−6^ relative peak absorbance for 1 %v H_2_ over 1 m of the absorption pathlength (1 %v∙m) under ambient conditions (Figure 2). Although such weak absorbance can be detected by an in situ TDLAS sensor, the line appears to be too weak for the sensor to detect H_2_ at sub-percentage levels (i.e., well below 1 %v∙m). However, it was found that simulations using the Voigt profile resulted in incorrect predictions in this particular case, because the Voigt profile is not adequate for modelling hydrogen absorption lines. 

It is important to note that, due to the low mass of the H_2_ molecule, Doppler broadening is significantly larger than what is typical for other molecules at this wavelength. The Doppler half width at half maximum (HWHM) for this transition is 0.0204 cm^−1^ at 296 K. The small collisional cross-section and absence of a dipole moment result in very weak collisional broadening, while the high rate of velocity-changing collisions contributes to unusually strong narrowing (the Dicke narrowing effect). The combination of these effects results in a line profile that deviated significantly from the Voigt profile. Hence, more advanced profiles must be used, such as the Hartmann–Tran profile (HTP) [25] or other simpler profiles that include collisional narrowing, such as the Rautian profile (RP) [26] and Galatry profile (GP) [27]. Recently non-Voigt line profiles have been implemented in the HITRAN database [28], where HTP was explicitly recommended for modelling the H_2_ lines. However, by the time of writing, the non-Voigt line parameters for H_2_ in air were not available in the HITRAN database. The line parameters used here for the S(1) (1-0) line for pure H_2_ (self-broadening) were reported by Wcisło et al. [28]. The reported self-broadening coefficient for this H_2_ line was $\gamma_{self}=$ 0.0019 cm^−1^atm^−1^, and the velocity-changing collision (narrowing) coefficient was $\nu_{self}^{vc}=$ 0.0448 cm^−1^atm^−1^. The combination of very weak self-broadening and strong narrowing should result in sub-Doppler widths of the H_2_ line for pressures around ambient air pressure. Indeed, significant reduction of the H_2_ linewidth with pressure and a corresponding increase in peak amplitude have been reported for all studied H_2_ lines in the overtone (2-0) vibrational band [6,24] for pure H_2_ gas. However, nitrogen and air broadening and narrowing parameters have not yet been reported. 

In this paper, the results of measuring the self-broadening and narrowing parameters and the corresponding parameters for nitrogen and air for the S(1) (1–0) line are presented. Since the laser intensity modulation amplitude, modulation phase, and phase shift between the laser intensity and wavelength modulations all influence the shape of the WMS absorption signals, performing WMS signal fitting using advanced line profiles with many fitting parameters can be ambiguous. Such an approach requires an exceptional signal-to-noise ratio and precision of the recorded WMS signals (better than 1% rel.), which is a very challenging task, especially in cases of weak H_2_ transition. Since such precision could not be achieved using the given sensor, no WMS signal fittings were performed in this study. Another approach was chosen based on findings by Reid and Labrie [29], who investigated the behavior of the 2f WMS lineshape as a function of the ratio between modulation amplitude a and absorption HWHM Δ: m=a/Δ. The WMS lineshapes for the Voigt profile and the limiting cases of the Voigt, Doppler, and Lorentzian profiles were modelled. It was found that the peak amplitude of the WMS lineshape (WMS-PA) was maximized at m= 2.2 independent of the ratio of collisional broadening to Doppler broadening (i.e., regardless of whether Voigt was in the Doppler or Lorentzian regime). To find out if the same value of m= 2.2 was also valid for an absorption lineshape exhibiting strong collisional narrowing, numerical simulations were performed for HTP, RP, and GP using the collisional broadening and narrowing parameters within the range of values reported for different H_2_ lines. In particular, the gas pressure region where collisional narrowing resulted in the most prominent effect on the H_2_ lineshape, between 0.1 and 1.0 atm, was of interest. 

The details of the RP and GP calculations can be found in the paper by Wang et al. [30]. HTP was calculated according to Forthomme et al. [31], who provide the Matlab implementation as a supplement to the paper.

The instantaneous laser frequency of the laser modulated at a frequency *f* around its center frequency v can be written as

(1) v(t)=v+acos(2πft)

The 2f WMS lineshape $WMS_{2f}\left(\nu ,a\right)$ in the limit of low absorbance, which is true in this case, is proportional to the second Fourier component of the absorbance [32,33]. Since the absorbance in turn is proportional to the line profile function $g\left(\nu_{d}\right)$, where $\nu_{d}$ is detuning from the absorption line center $\nu_{0}$ ($\nu_{d}=v-\nu_{0}$), the 2f WMS lineshape can be calculated from the following integral:

(2) $$WMS_{2f}\left(\nu_{d},a\right)\propto \frac{1}{\tau }\int_{0}^{\tau }g\left(v_{d}+a\cos\left(2\pi ft\right)\right)\cos\left(2\pi 2ft\right)dt$$

where *τ* is an integration time, $\tau \gg f^{-1}$.

In the modelling of the WMS lineshapes using different line profiles, the line profile function (e.g., HTP) was substituted into Equation (2), and the integral was calculated numerically.

**Figure 4:**
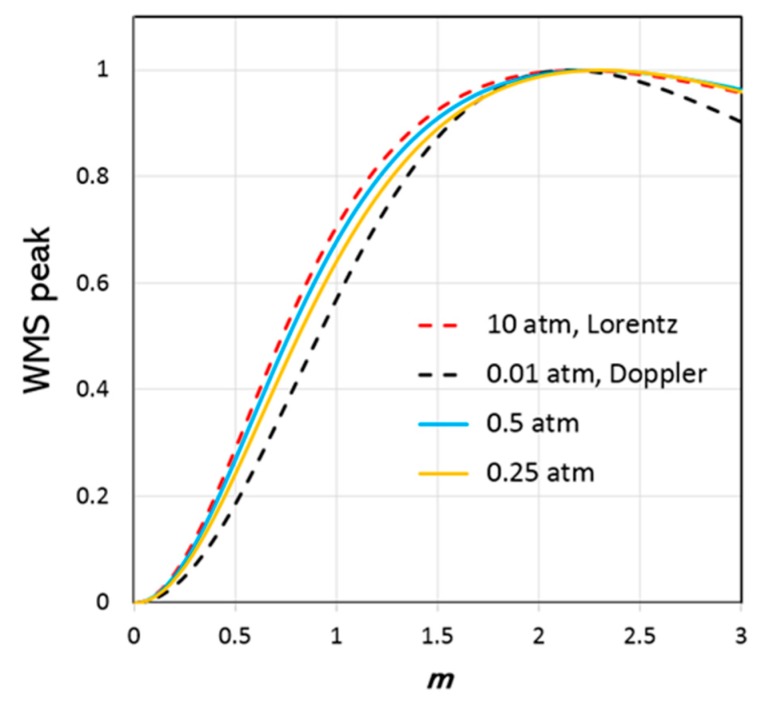
Calculated wavelength modulation spectroscopy (WMS) peak amplitude (Hartmann–Tran profile (HTP)) as a function of normalized modulation amplitude *m* for H_2_ at and 0.25 and 0.5 atm (100% H_2_) together with the Lorentzian limit at 10 atm and the Doppler limit at 0.01 atm. HTP line parameters for S(1) (1–0) H_2_ transition given in [28] were used in the modelling.

The modelling shows that the peak value for WMS-PA appears to be approximately at the same value of m= 2.2 ± 0.1 in each case of HTP, RP, and GP used in the WMS lineshape. Figure 4 shows the results of modelling the S(1) (1–0) H_2_ line using HTP and the line parameters for 100% H_2_ gas by Wcisło et al. The results for the Doppler profile were obtained by setting the H_2_ pressure to P= 0.01 atm, which ensures that both collisional broadening and narrowing effects are negligible. The results for the Lorentzian profile were obtained by setting P= 10 atm, which ensures that both Doppler broadening and collisional narrowing are negligible. The plots for P= 0.25 atm and P= 0.5 atm represent the region where collisional narrowing is significant. No significant differences between the WMS-PA *m*-dependence for HTP, RP, and GP were found. This means that it is possible to indirectly measure the HWHM of an absorption line by varying the modulation amplitude of the laser while measuring the WMS-PA. Finally, the HWHM pressure dependence obtained from the measurements can be compared with the corresponding theoretical pressure dependence from the model. Such comparison should provide a good estimation of the collisional broadening and narrowing coefficients (with accuracy of the parameters determined by this method of about 10% rel.).

To improve the SNR of the WMS-PA measurement, the sensor was connected to a Herriott-type multipass cell with 12 m pathlength, which was filled with pure H_2_ gas at different pressures up to 4 atm. For measurement in nitrogen and air gas mixtures, pure hydrogen was diluted to 10 %v in the corresponding balance gas using a HovaGAS G6 (IAS GmbH) gas mixer. The Doppler HWHM at 23 °C of 0.204 cm^−1^ was used to calibrate the laser modulation amplitude *a* to the unit cm^−1^ such that the experimentally obtained HWHM converged to the theoretical Doppler HWHM at zero pressure. The theoretical HWHM was obtained from the calculated HTP for different pressures. The results from the calculations were compared with the measured values, and the broadening and narrowing coefficients used in the calculations were adjusted until the best fit with the measured results was achieved (ignoring line shift effects). It should be noted that accuracy of the experimental data (measured WMS-PA as a function of *m*) did not allow us to distinguish between HTP, RP, and GP. The calculated HWHM using any of these three profiles showed very similar pressure dependence, and the obtained broadening and narrowing parameters were essentially the same, while HTP was used solely to comply with the new recommendation for the H_2_ absorption lines adopted in HITRAN. The parameter for broadening speed dependence had a negligible influence on the resulting pressure dependency of the HWHM. In the modelling, this parameter was set to one-tenth of the broadening parameter, and the correlation parameter was set to zero.

**Figure 5:**
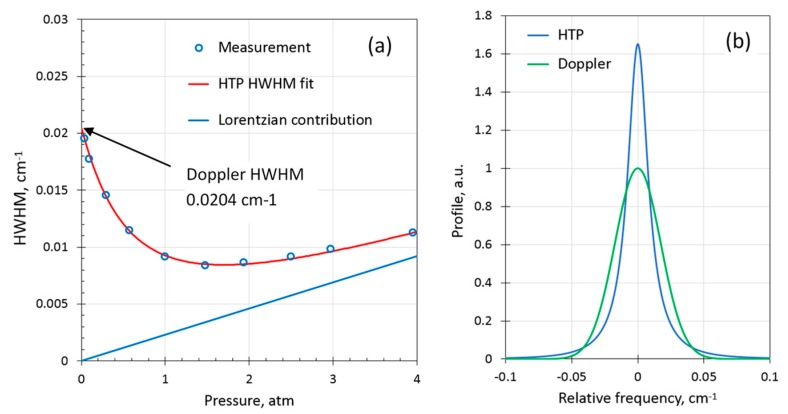
(**a**) Half width at half maximum (HWHM) of H_2_ 100 %v as a function of pressure together with a fit using the HTP; (**b**) calculated HTP at *P =* 1.0 atm compared to the corresponding Doppler profile.

Figure 5a shows the measured HWHM using the WMS-PA approach of the H_2_ line for pure (100 %v) H_2_ gas and the calculated HTP HWHM that resulted in the best fit. The coefficients obtained were $\gamma_{self}=$ 0.0024(3) cm^−1^atm^−1^ and $\nu_{self}^{vc}=$ 0.037(5) cm^−1^atm^−1^ (*T* = 296 K). These coefficients are in reasonable agreement with the values reported by Wcisło et al.: 0.0019(1) and 0.0448, respectively (reported for *T* = 315 K). As seen in Figure 5a, the absorption line of pure H_2_ is extremely narrow (more than twice as narrow as the Doppler HWHM) in a broad pressure range from around ambient up to several atm. For demonstration purposes, Figure 5b compares HTP calculated for 1 atm using the obtained parameters with the Doppler profile of the same integral. The peak amplitude of the H_2_ line (100%) at atmospheric pressure appears to be more than a factor of 1.6 larger than if the H_2_ line were pure Doppler (i.e., without any collisional broadening at all).

**Figure 6:**
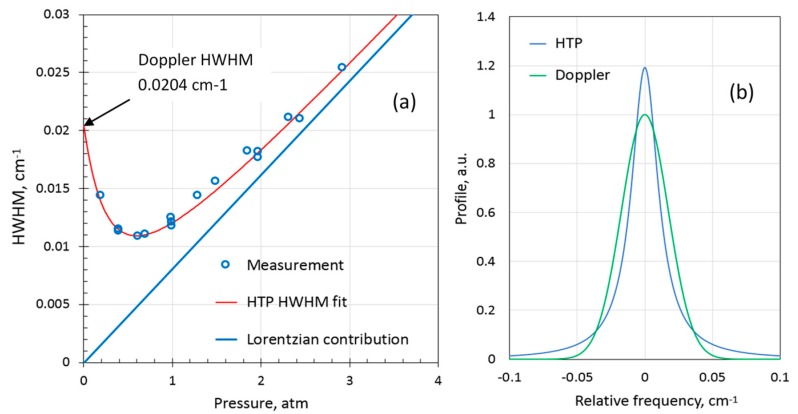
(**a**) HWHM of 10 %v H_2_ in N_2_ balance gas as function of pressure and a fit using the HTP; (**b**) calculated HTP at *P =* 1.0 atm compared to the corresponding Doppler profile.

For gas sensing, it is important to measure the collisional parameters (broadening and narrowing) in nitrogen and air balances. These parameters are not available in the literature for the given H_2_ transition. Figure 6a shows the pressure dependence of measured H_2_ HWHM for 10% H_2_ in nitrogen balance and the best fit of the calculated HTP HWHM. Despite significantly larger nitrogen broadening compared to the self-broadening, the H_2_ line in nitrogen around ambient pressures is narrower than the Doppler broadened line. In Figure 6b, the modelled HTP at 1.0 atmosphere is compared with the Doppler profile of the same integral. The peak amplitude of the H_2_ line at 1 atm in nitrogen is 1.2 times larger than if the line had only Doppler broadening. The collisional broadening and narrowing coefficients for nitrogen balance were evaluated to be $\gamma_{N2}=$ 0.0087(8) cm^−1^atm^−1^ and $\nu_{N2}^{vc}=$ 0.071(7) cm^−1^atm^−1^, respectively (*T* = 296 K). A few measurements of H_2_ in air balance at ambient pressure were made as well. The H_2_ line appeared somewhat narrower than in nitrogen balance. It was assumed that the collisional narrowing parameter was the same as in nitrogen. The broadening coefficient in air was then estimated to be $\gamma_{AIR}=$ 0.0081(8) cm^−1^atm^−1^. Table 1 summarizes the results for the 2122 nm H_2_ line obtained in this study.

## 5. H_2_ Sensor Performance

The simulation using HTP with the line strength listed in HITRAN and the collisional parameters obtained in this study for H_2_ in nitrogen and air indicates that 1 %v H_2_ over a 1-m optical pathlength (1 %v∙m) gives about 2.2 × 10^−5^ of relative peak absorbance. This is about four times stronger than the absorbance calculated using the Voigt profile with default HITRAN parameters. To achieve the required LOD of 0.1–0.2 %v∙m (assuming a measurement range of 0–10 %v), the sensitivity of the TDLAS sensor must be in the range of 2 to 4 × 10^−6^ of relative absorbance. The two main factors limiting the sensitivity of most TDLAS sensors are (i) optical fringe-noise, sometimes called etalon noise, which is interference of the laser light caused by partially reflective surfaces in the system, and (ii) coupling of stray light into the active laser area (laser feedback noise). Considerable effort was made in the optomechanical design of the H_2_ sensor to eliminate most sources of optical feedback and etalon noise. All optical components used were wedged and tilted and all optical surfaces AR coated. The optical system was designed in a way to avoid distances that could create etalon fringes with periods close to the H_2_ absorption width. In addition, the modulation amplitude was carefully optimized to improve sensitivity to and selectivity of the H_2_ absorption. The H_2_ line is approximately five times narrower than the potentially interfering lines of CO_2_, CH_4_, and NH_3_. If the modulation amplitude is optimized for the H_2_ line (1 atm, N_2_/air) using *m* = 2.2, the corresponding value of *m* for the interfering lines will be around 0.4–0.5, such that the WMS signals for these lines are efficiently suppressed by approximately four times (see Figure 4). The modulation amplitude can be further adjusted down to *m* = 1.5 for the H_2_ line without significant reduction of H_2_ WMS-PA (0.9 of the maximum WMS-PA), while the *m* value for the interfering lines reduces to about 0.3 (about 0.15 of the maximum WMS-PA), or an additional two-times reduction of the corresponding WMS-PA. In total, such optimization of the modulation amplitude provides about seven times (4 × 0.9 × 2) suppression of the interference signal. Thus, the WMS technique is particularly useful for discrimination between absorption features of different widths, which is a great advantage over DAS, especially when it comes to sensing hydrogen. A notable byproduct of relatively weak laser wavelength modulation is that the residual amplitude modulation contributing to baseline variations in the WMS signal [34] becomes negligible, thus improving detection sensitivity.

The sensor was calibrated with a known concentration of H_2_ in N_2_ balance and characterized for different pressures and temperatures by measuring pressure and temperature dependencies of the H_2_ WMS signal using a heated cell. For evaluation of the sensor performance, the transmitter and receiver were set 1 m apart on a test bench. A 0.7-m optical cell fitted with wedged windows was placed between the transmitter and the receiver. Gas mixtures were directed into the cell at flow rates from 1 to 5 L/min. The pressure and temperature of the cell were close to ambient. For long-term stability tests, the cell was filled with a known gas mixture and sealed. The results were converted to the gas concentration unit, %v∙m. For zero H_2_ measurement, the cell was removed from the test bench and ambient air was measured. 

**Figure 7:**
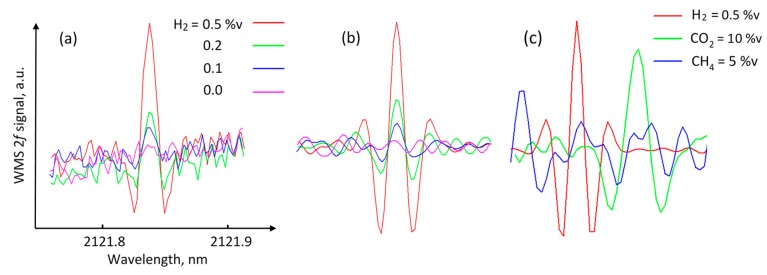
(**a**) 2f WMS sensor signals for three H_2_ concentrations and one zero signal. Corresponding concentrations at 1 m of optical pathlength are 0.5, 0.2, and 0.1 %v. Zero signal was recorded in ambient air. (**b**) Signals after convolution with matched Mexican hat wavelet function demonstrating improved SNR. (**c**) Convolved 2f WMS signals of 0.5 %v of H_2_, 10 %v CO_2_, and 5 %v CH_4_.

Figure 7a shows the obtained 2f WMS signals for 0.5, 0.2, and 0.1 %v of H_2_ in nitrogen for an optical pathlength of 1 m. The signal for zero hydrogen concentration was recorded when the sensor was measured in ambient air. Each signal was acquired during 1 s as a result of averaging 150 laser wavelength scans. To improve sensitivity, the signals can be further processed using, for example, wavelet denoising or band-pass filtering. Here, convolution with a Mexican hat wavelet function was used. The width of the function was chosen to match the width of the H_2_ absorption feature. Figure 7b demonstrates the improved SNR after convolution. The baseline slope and high-frequency noise components were removed. It is seen that the peak from 0.1 %v∙m is clearly distinguishable from the noise level, which indicates that the obtained sensitivity of the sensor to fractional absorbance was better than 2 × 10^−6^. The SNR approach was applied to estimate the LOD of the sensor. In this approach, LOD corresponds to the concentration level at which the measured signal (peak-to-peak) reaches three times the signal noise (peak-to-peak) of the baseline. By using this criterion, the LOD of the H_2_ sensor was estimated to be 0.1 %v∙m for the 1-s integration time. Figure 7c demonstrates the selectivity of the sensor, showing the convolved 2f WMS signals of 0.5 %v of H_2_, 10 %v of CO_2_, and 5 %v CH_4_ (all in nitrogen balance) recorded at 1 atm pressure. It should be mentioned that as the pressure increases, the selectivity improves, because the *m* value for the interfering lines decreases at a much higher rate than the *m* value of the H_2_ line due to the significant difference in pressure-broadening parameters. 

**Figure 8:**
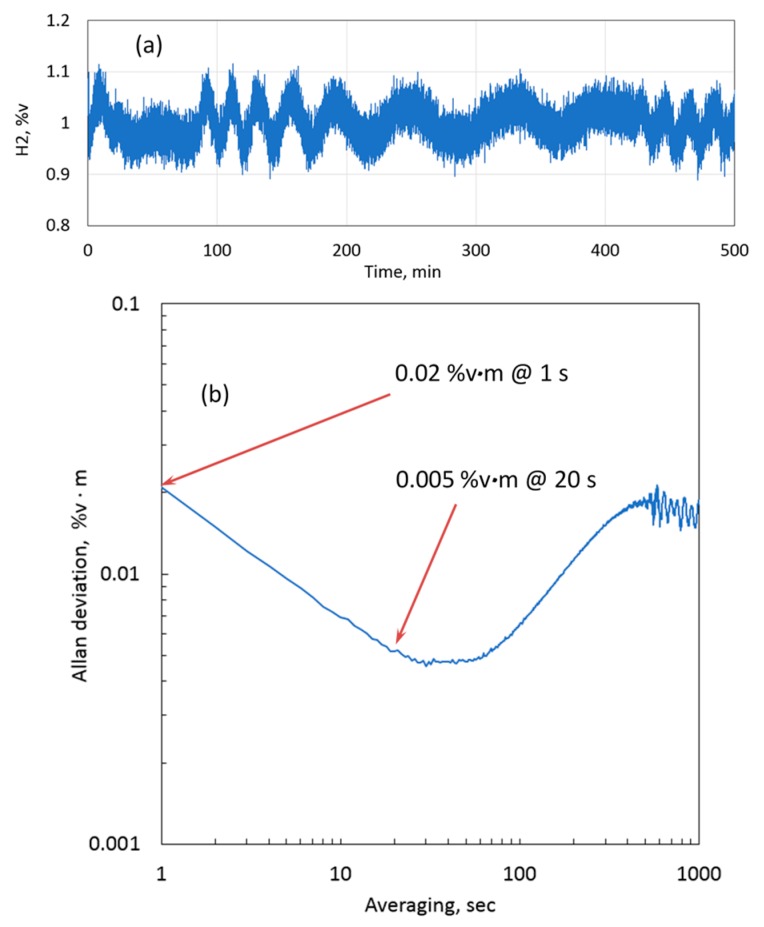
(**a**) Long-term measurement of 1 %v H_2_ over 1 m with a 1-s update time; (**b**) Allan deviation of sensor measurements (1σ). Demonstrated precision is 0.02 %v∙m at 1 s with no averaging and 0.005 %v∙m with 20 s averaging.

To examine the capability of the sensor in terms of measurement precision and long-term stability, the sensor was installed on a cell to measure 1.0 %v∙m H_2_ in nitrogen, and the data were logged over 500 min with a 1-s resolution. Figure 8a shows the plot of the measured concentration. Along with stochastic noise, an oscillating pattern can be observed. This pattern was attributed to optical fringes from the cell windows, which were drifting due to the changing ambient temperature. The corresponding Allan deviation (1σ) [35] is plotted in Figure 8b. The precision without averaging (i.e., with a sensor update time of 1 s) was 0.02 %v∙m. The precision improved with averaging, and after 20 s of averaging 0.005 %v∙m was achieved. Between 1 and 10 min of averaging time, the Allan deviation increased up to approximately 0.02 %v∙m. This behavior can be attributed to a combination of etalon noise from the cell windows and the sensor optical system. Thus, long-term drift and short-term (1 s) precision were both at about the same value of 0.02 %v∙m, which demonstrates excellent overall performance of the sensor. 

**Figure 9:**
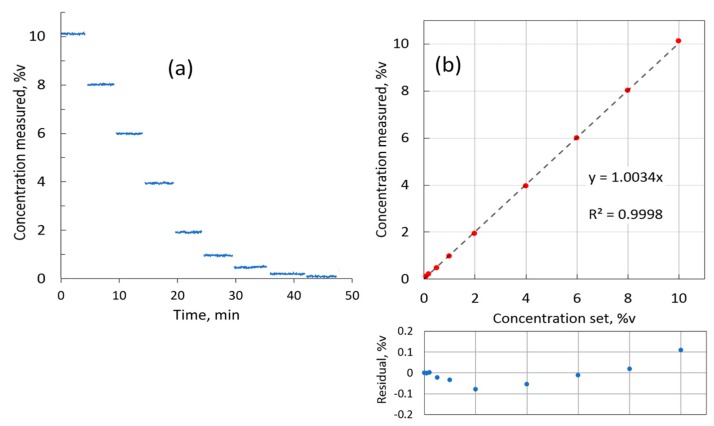
(**a**) Sensor response to different H_2_ concentrations (10.0, 8.0, 6.0, 4.0, 2.0, 1.0, 0.5, 0.2, 0.1 %v) in the 0.7 m cell; (**b**) plot of measured concentration as a function of concentration set in gas mixer and linear fit.

For linearity evaluation, the HovaGAS gas mixer was used to generate H_2_ concentrations stepwise down from 10 to 0.1 %v by diluting pure hydrogen in nitrogen base. Each concentration was measured for about 4 min. Figure 9a shows the recorded measurements. The measurements for each concentration step were averaged and plotted in Figure 9b against the nominal concentration set in the gas mixer software. The linear fit has a slope well within 0.5% of the sensor range, and the *R*-square value of 0.9998 confirms the excellent linearity of the H_2_ sensor. 

## 6. Conclusions

A TDLAS sensor was developed for industrial in situ and noncontact measurement of hydrogen. The sensor uses a well-proven LaserGas II platform by NEO Monitors AS and a 2122-nm DFB laser. The selected quadrupole absorption line of H_2_ was characterized in terms of collisional broadening and narrowing effects. The line exhibited sub-Doppler width in wide pressure ranges not only for pure hydrogen but also for hydrogen in nitrogen and air balances. The broadening and narrowing coefficients for nitrogen and air were obtained by fitting the measured linewidth to the calculated linewidth from the Hartmann–Tran profile. The performance of the sensor was evaluated. Measurement precision of 0.02 %v H_2_ for just 1 m of the absorption pathlength was achieved for 1 s of integration time. Using the SNR approach, the corresponding LOD was estimated to be 0.1 %v of H_2_ in air, which is sufficient for safety applications. By increasing the absorption pathlength and/or integration time, the performance of the sensor can be further improved, which should widen its applicability to industrial processes with sub-% H_2_ levels.

## Figures and Tables

**Table 1:** H_2_ 2122 nm line parameters obtained in this study (*T* = 296 K); unit is cm^−1^atm^−1^.

	γ	$\nu^{vc}$
H_2_–N_2_	0.0087(8)	0.071(7)
H_2_–air	0.0081(8)	− ^1^
H_2_–H_2_	0.0024(3)	0.037(5)
H_2_–H_2_^2^	0.0019(1)	0.0448

^1^ Assumed to be the same value as for N_2_. ^2^ Results for *T* = 315 K reported by Wcisło et al. [28]
